# Long-COVID Headache

**DOI:** 10.1007/s42399-021-00964-7

**Published:** 2021-05-20

**Authors:** Paolo Martelletti, E. Bentivegna, V. Spuntarelli, M. Luciani

**Affiliations:** 1 Department of Clinical and Molecular Medicine, Sapienza University, Rome, Italy; 2 Emergency Medicine and Regional Referral Headache Centre, Sant’Andrea Hospital, Rome, Italy; 3 Residency Program, Internal Medicine, Sapienza University of Rome, Rome, Italy; 4 COVID-19 Unit, Santo Spirito Hospital, Rome, Italy; 5 COVID-19 Emergency Unit, Sant’Andrea Hospital, Rome, Italy; 6 Internal Medicine Unit, Belcolle Hospital, Viterbo, Italy

**Keywords:** Long-Covid-19, Peristent headache, Neurological sequalae, Pain, Classification

## Abstract

The so-called long COVID-19 is a set of symptoms that accompanies the patient even for months after discharge from the hospital. These symptoms include easy muscle fatigue, moderate breathlessness, persistent headache, the feeling of a foggy head, and the development of psychiatric disorders. In general, the quality of life of at least half of the patients who come out of the COVID-19 syndrome, both mild and severe, shows a markedly worsening despite having passed a difficult physical and psychological test. Among all the neurological disorders that can most frequently be found in the long COVID-19, it is important to consider the persistent headache symptomatology as a possible chronic sequela of the infection. Since there is not a definition in the International Headache Society classification of this type of headache, we must focus our attention on this long-COVID-19 headache especially because clinical studies are being planned to collect big data for the International Headache Society Classification Committee.

Headache occurs as one of the initial symptoms of infection in COVID-19. It can be spread throughout the skull, giving a feeling of constriction and weight at the top of the head. All this occurs in a large part of subjects who present the first symptoms of COVID-19 in a percentage that varies from 14 to 60% [1–3]. If the infection affects a subject already suffering from migraine, it multiplies the crises and makes recognition not immediate, together with the fever, fatigue, muscle weakness, and respiratory difficulty of the infection [1].

The symptomatology of COVID-19 infection is multifaceted and depends on the involvement of multiple systems and apparatuses, not just the pulmonary one [4]. In non-severe forms of COVID-19, that can and must be managed and treated by avoiding the hospitalization of the patient, early recognition of symptoms is a fundamental prerequisite for immediate therapeutic management [5].

The so-called long COVID is the set of symptoms that accompanies the patient even for months after discharge from the hospital. These symptoms include easy muscle fatigue, moderate breathlessness, persistent headache, the feeling of a foggy head, and the development of psychiatric disorders [6]. In general, the quality of life of at least half of the patients who come out of the COVID-19 syndrome, both mild and severe, shows a markedly worsening despite having passed a difficult physical and psychological test [7].

The neurological impairment of post-COVID-19 can have different pathophysiological bases: direct neuro-invasion with a damage on the neuronal pathway, indirect effects mediated by hypoxia, hypertension, coagulopathy and cytokine storm on the CNS, up to the worsening of pre-existing brain diseases or new ones (cerebrovascular events, infectious and toxic encephalopathy, meningoencephalitis, Guillain Barré syndrome) [8]. Long-term complications arise from this multifaceted picture.

Among all the neurological disorders that can most frequently be found in the “long COVID-19,” it is not necessary to underestimate the persistent headache for at least 6 months, both as a clinical expression of new onset concomitant with cognitive blunting, such as “brain fog,” and as worsening/chronicization of a pre-existing migraine [9]. Although headache does not represent a prognostic picture for the evolution of COVID-19, it must always be taken into consideration as a possible chronic sequela of the infection.

A quite similar clinical picture of sub-continuous headache was already described, as a consequence of other viral infections, as the New Daily Persistent Headache (NDPH), and had been described by Diaz-Mitoma and Walter Vanast of McGill University in Montreal, as headache resulting from Esptein-Barr Virus infection and published in 1987 in Lancet [10], headache syndrome lasting >3 months. However, this putative similarity between NDPH and long-COVID headache needs further discussion and data.

In this scientific moment of a not yet incomplete consensus not only on the nosography classification of the sequelae of COVID-19, namely long-COVID, post-acute sequelae of SARS-COV-2 (PASC, COVID long haulers, etc.), the presence of this multifaceted neurological symptomatology, headache and foggy feeling, even after negativization by COVID-19 infection, must be taken into serious consideration in order to avoid a chronicization of the headache and a further worsening of the patient’s quality of life [11–17]. Furthermore, deranged innate immune signaling and activation of inflammasomes implicated in both COVID-19 headache and migraine [18] could also play a role in long-COVID headache, as well as sleep difficulties associated with long-COVID symptoms also be a significant element determining cognitive problems, poor memory, and chronicity of headaches [19].

## Data Availability

Not applicable.

## References

[1] Martelletti P, Bentivegna E, Luciani M, Spuntarelli V (2020). Headache as a prognostic factor for COVID-19. Time to re-evaluate. SN Compr Clin Med.

[2] Howard LM, Garguilo K, Gillon J, Seegmiller AC, Schmitz JE, Webber SA, Banerjee R. Characteristics and clinical features of SARS-CoV-2 infections among ambulatory and hospitalized children and adolescents in an integrated health care system in Tennessee. medRxiv [Preprint]. 2020 13:2020. 10.1101/2020.10.08.20208751.

[3] Pullen MF, Skipper CP, Hullsiek KH, Bangdiwala AS, Pastick KA, Okafor EC, Lofgren SM, Rajasingham R, Engen NW, Galdys A, Williams DA, Abassi M, Boulware DR (2020). Symptoms of COVID-19 outpatients in the United States. Open Forum Infect Dis.

[4] Spuntarelli V, Luciani M, Bentivegna E, Marini V, Falangone F, Conforti G, Rachele ES, Martelletti P (2020). COVID-19: is it just a lung disease? A case-based review. SN Compr Clin Med.

[5] Pennica A, Conforti G, Falangone F, Martocchia A, Tafaro L, Sentimentale A, Marini V, Pezzuto A, Spuntarelli V, Martelletti P (2020). Clinical management of adult coronavirus infection disease 2019 (COVID-19) positive in the setting of low and medium intensity of care: a short practical review. SN Compr Clin Med.

[6] Taquet M, Geddes JR, Husain M, Luciano S, Harrison PJ (2021). 6-month neurological and psychiatric outcomes in 236 379 survivors of COVID-19: a retrospective cohort study using electronic health records. Lancet Psychiatry.

[7] Willi S, Lüthold R, Hunt A, Hänggi NV, Sejdiu D, Scaff C, Bender N, Staub K, Schlagenhauf P (2021). COVID-19 sequelae in adults aged less than 50 years: a systematic review. Travel Med Infect Dis.

[8] Abboud H, Abboud FZ, Kharbouch H, Arkha Y, El Abbadi N, El Ouahabi A (2020). COVID-19 and SARS-Cov-2 infection: pathophysiology and clinical effects on the nervous system. World Neurosurg.

[9] Mendelson M, Nel J, Blumberg L, Madhi SA, Dryden M, Stevens W, Venter FWD (2020). Long-COVID: an evolving problem with an extensive impact. S Afr Med J.

[10] Diaz-Mitoma F, Vanast WJ, Tyrrell DL (1987). Increased frequency of Epstein-Barr virus excretion in patients with new daily persistent headaches. Lancet.

[11] Mahajan N, Singla M, Singh B, Sajja V, Bansal P, Paul B, Goel P, Midha R, Bansal R, Singh G (2020). 2019-NCoV: What every neurologist should know?. Ann Indian Acad Neurol.

[12] Peluso MJ, Kelly JD, Lu S, Goldberg SA, Davidson MC, Mathur S, Durstenfeld MS, Spinelli MA, Hoh R, Tai V, Fehrman EA, Torres L, Hernandez Y, Williams MC, Arreguin MI, Bautista JA, Ngo LH, Deswal M, Munter SE, Martinez EO, Anglin KA, Romero MD, Tavs J, Rugart PR, Chen JY, Sans HM, Murray VW, Ellis PK, Donohue KC, Massachi JA, Weiss JO, Mehdi I, Pineda-Ramirez J, Tang AF, Wenger M, Assenzio M, Yuan Y, Krone M, Rutishauser RL, Rodriguez-Barraquer I, Greenhouse B, Sauceda JA, Gandhi M, Hsue PY, Henrich TJ, Deeks SG, Martin JN. Rapid implementation of a cohort for the study of post acute sequelae of SARS-CoV-2 infection/COVID-19. medRxiv [Preprint]. 2021 12:2021.03.11.21252311. 10.1101/2021.03.11.21252311.

[13] Fernández-de-Las-Peñas C, Palacios-Ceña D, Gómez-Mayordomo V, Cuadrado ML, Florencio LL (2021). Defining post-COVID symptoms (post-acute COVID, long COVID, persistent post-COVID): an integrative classification. Int J Environ Res Public Health.

[14] Meagher T. Long COVID - an early perspective. J Insur Med. 2021 30. 10.17849/insm-49-1-1-5.1. Online ahead of print. 10.17849/insm-49-1-1-5.1 33784738

[15] Graham EL, Clark JR, Orban ZS, Lim PH, Szymanski AL, Taylor C, DiBiase RM, Jia DT, Balabanov R, Ho SU, Batra A, Liotta EM, Koralnik IJ (2021). Persistent neurologic symptoms and cognitive dysfunction in non-hospitalized Covid-19 “long haulers”. Ann Clin Transl Neurol.

[16] Nalbandian A, Sehgal K, Gupta A, Madhavan MV, McGroder C, Stevens JS, Cook JR, Nordvig AS, Shalev D, Sehrawat TS, Ahluwalia N, Bikdeli B, Dietz D, Der-Nigoghossian C, Liyanage-Don N, Rosner GF, Bernstein EJ, Mohan S, Beckley AA, Seres DS, Choueiri TK, Uriel N, Ausiello JC, Accili D, Freedberg DE, Baldwin M, Schwartz A, Brodie D, Garcia CK, Elkind MSV, Connors JM, Bilezikian JP, Landry DW, Wan EY. Post-acute COVID-19 syndrome. Nat Med. 2021 22. 10.1038/s41591-021-01283-z. Online ahead of print. 10.1038/s41591-021-01283-z PMC8893149 33753937

[17] Huang Y, Pinto MD, Borelli JL, Mehrabadi MA, Abrihim H, Dutt N, Lambert N, Nurmi EL, Chakraborty R, Rahmani AM, Downs CA. COVID symptoms, symptom clusters, and predictors for becoming a long-hauler: looking for clarity in the haze of the pandemic. medRxiv. 2021 5:2021.03.03.21252086. 10.1101/2021.03.03.21252086. 10.1177/10547738221125632 PMC9510954 36154716

[18] Bolay H, Gül A, Baykan B (2020). COVID-19 is a real headache!. Headache.

[19] Huang C, Huang L, Wang Y, Li X, Ren L, Gu X, Kang L, Guo L, Liu M, Zhou X, Luo J, Huang Z, Tu S, Zhao Y, Chen L, Xu D, Li Y, Li C, Peng L, Li Y, Xie W, Cui D, Shang L, Fan G, Xu J, Wang G, Wang Y, Zhong J, Wang C, Wang J, Zhang D, Cao B (2021). 6-month consequences of COVID-19 in patients discharged from hospital: a cohort study. Lancet.

